# Phase II study on the combination of irinotecan plus cisplatin as a second-line therapy in patients with advanced or recurrent gastric cancer

**DOI:** 10.3892/mco.2013.115

**Published:** 2013-05-09

**Authors:** YASUSHI RINO, NORIO YUKAWA, TSUTOMU SATO, TAKASHI OSHIMA, HIROYASU TANABE, YUJI YAMAMOTO, HIROSHI MATSUKAWA, RYUJI SHIRAISHI, TOSHIO IMADA, MUNETAKA MASUDA

**Affiliations:** 1 Department of Surgery, School of Medicine, Kanazawa-ku, Yokohama 236-0004;; 2 Department of Gastroenterology, Medical Center, Yokohama City University, Minami-ku, Yokohama 232-0024;; 3 Department of Surgery, Kamishirane Hospital, Asahi-ku, Yokohama 241-0002;; 4 Department of Surgery, Kanagawa Prefectural Ashigarakami Hospital, Ashigarakami-gun, Kanagawa 258-0003;; 5 Department of Surgery, Yokohama Minami Kyosai Hospital, Kanazawa-ku, Yokohama 236-0037;; 6 Department of Surgery, Hiratsuka Kyosai Hospital, Hiratsuka, Kanagawa 254-0047;; 7 Saiseikai Yokohamasi Nanbu Hospital, Konan-ku, Yokohama 234-0054, Japan

**Keywords:** advanced gastric cancer, recurrent gastric cancer, phase II study, second-line chemotherapy, cisplatin, irinotecan

## Abstract

A pilot phase II study was conducted to evaluate the efficacy and safety of the combined administration of irinotecan (CPT-11) plus cisplatin (CDDP) as a second-line therapy for advanced or recurrent gastric cancer. Between November, 2006 and May, 2009, 18 patients were enrolled in this study. The patients were required to have received prior chemotherapy with S-1 (n=17), an orally administered 5-fluorouracil (5-FU) prodrug, or S-1 plus CDDP (n=1). CPT-11 and CDDP were administered at a dose of 60 and 30 mg/m^2^, respectively, on days 1 and 15 of a 4-week treatment cycle. The regimen was repeated until the occurrence of unacceptable toxicity, disease progression, or patient refusal. The primary endpoint of this study was the response rate (RR). In the second-line setting, 2 cases of complete response (CR), 1 of partial response (PR) and 7 of stable disease (SD) were identified. The RR was 16.7% and the disease control rate (DCR) was 55.6%. The median survival time (MST) and progression-free survival (PFS) was 282 and 111 days, respectively. As regards hematological toxicity, the major adverse effect during the second-line of chemotherapy was grade 3–4 leukopenia (22.2%). In addition, with regard to non-hematological toxicities, the major adverse effect during the second-line chemotherapy was grade 3–4 loss of appetite (11.1%). There was no mortality attributable to the adverse effects of the drugs. Findings of the present study suggested that CPT-11 and CDDP combination therapy in a second-line setting is an effective regimen in the treatment of advanced gastric cancer.

## Introduction

Previous clinical trials have demonstrated the efficacy of certain chemotherapeutic agents against gastric cancer (1,2). S-1 or S-1 plus cisplatin (CDDP) combination chemotherapy have gradually been established as the front-line chemotherapeutic agent in Japan for the treatment of unresectable, resected but not cured, or recurrent gastric cancer (3). However, certain types of gastric cancer do not respond to this agent. Therefore, in the cases where S-1 therapy was unsuccessful, a second-line regimen was administered using or adding other agents such as CDDP, paclitaxel (PTX), docetaxel (DTX) and irinotecan (CPT-11). When a patient administered S-1 exhibited progressive disease, treatment with CDDP, PTX, DTX or CPT-11 without cross-tolerance of S-1 resulted in a good outcome.

However, an effective second-line chemotherapy for advanced and recurrent gastric cancer has yet to be established. Previously, we administered S-1 alone or S-1 plus CDDP combination chemotherapy as the first-line and CPT-11 plus CDDP as the second-line chemotherapy regimen. The CPT-11 plus CDDP regimen had a high response rate (RR) of >53.5% in patients without prior chemotherapy and it was also reported that neutropenia grade 3 or higher accounted for <40% of the cases (4). Therefore, it was concluded that curative effects may be expected even after the administration of S-1 or S-1 plus CDDP. However, there is no evidence that clearly demonstrates the effectiveness of the CPT-11 plus CDDP regimen following the failure of S-1 or S-1 plus CDDP.

We conducted a phase II study to evaluate the effectiveness of CPT-11 plus CDDP as a second-line chemotherapeutic regimen, following S-1 or S-1 plus CDDP therapy, in the treatment of advanced gastric cancer, by measuring the objective RR, the time to progression, the overall survival (OS) and the safety profile.

## Patients and methods

### Patient eligibility

A total of 18 patients with unresectable advanced or recurrent gastric cancer were enrolled in this study between November, 2006 and May, 2009. Eligibility criteria included histologically or cytologically confirmed gastric adenocarcinoma that was either unresectable (n=7) or recurrent (n=11) and the presence of measurable lesions. The patients were required to have received prior S-1 (n=17) or S-1 plus CDDP (n=1) chemotherapy (Table I).

Recurrent patients were included if at least 24 weeks had elapsed after the last postoperative S-1 or S-1 plus CDDP adjuvant chemotherapy. The patients were also required to meet the following criteria: age <75 years, amenability to oral administration of drugs, a Karnofsky performance score of ≥60, a life expectancy of ≥3 months and an adequate hematological status (defined as a total leukocyte count of >3,500/mm^3^, neutrophil count of >1,500/mm^3^, platelet count of >100,000/mm^3^, serum creatinine <1.5 mg/dl, total serum bilirubin <1.5 mg/dl, AST and ALT levels <2 times the upper limit of the normal range). Patients were excluded from the study in the case of concurrent or prior malignancies, active uncontrolled infections or other diseases, or a neurological or mental disease that prevented adequate comprehension of information. The pretreatment evaluation consisted of a complete history and physical examination, blood count, serum biochemistry and computed tomography (CT) of the chest and abdomen. The patients provided informed consent prior to the initiation of the treatment. This study was approved by the Ethics Committee of each participating institution.

### Study design

The S-1 first-line chemotherapy regimen was as follows: S-1 was administered orally twice daily following breakfast or dinner, at 80 mg/m^2^/day for 4 weeks, followed by 2 weeks of rest. During the course of the treatment, the patients had a complete blood count (CBC), biochemical and physical examinations every 2 weeks and the presence of tumor markers (CEA, CA19-9, STn and SLX) was assessed every 4 weeks. The treatment response was then evaluated by CT every 2 months.

The S-1 plus CDDP first-line chemotherapy regimen was as follows: S-1 was administered at the same dose as described above for 3 weeks, followed by 2 weeks of rest. On day 8, S-1 was combined with CDDP at a dose of 60 mg/m^2^. The patients were premedicated with 8 mg dexamethasone and 10 mg azasetron hydrochloride diluted in 50 ml of saline, given intravenously (i.v.) 30 min prior to treatment. Subsequently, 60 mg/m^2^ of CDDP was administered by i.v. infusion over 120 min. During the course of the treatment, the patients had a CBC, biochemical and physical examinations every 2–3 weeks and were assessed for tumor markers every 4 weeks. The treatment response was evaluated by CT every 2 months.

The regimen was modified in the case of >grade 3 toxicity, disease progression and elevated tumor markers. However, if grade 3–4 toxicity was observed after the first-line chemo-therapy, the second-line chemotherapy was administered after the patient recovered from the toxicity.

The second-line chemotherapy regimen was as follows: CPT-11 and CDDP were administered at a dose of 60 and 30 mg/m^2^, respectively, on days 1 and 15 of a 4-week treatment cycle. Prior to the administration, the patients were administered 10 mg azasetron hydrochloride and 8 mg dexamethasone i.v., with 100 ml saline water over 30 min. CPT-11 was administered by i.v. infusion at a dose of 60 mg/m^2^ with 500 ml saline water over 90 min and CDDP was administered by i.v. infusion at a dose of 30 mg/m^2^ with 500 ml saline water over 90 min.

The i.v. treatments were performed on an outpatient basis. The treatment was discontinued in the case of any ≥grade 3 hematological or non-hematological toxicity or at the request of the patients and an alternative third-line chemotherapy was then performed.

### Study evaluations

The responses were assessed by physical examination, direct visualization, examination of the upper gastrointestinal tract following a barium meal, gastrofibroscopy and CT. Tumor evaluation was performed every 2 months according to the World Health Organization criteria and the responses were confirmed by radiography within 2 weeks. A complete response (CR) was defined as remission of the disease for a minimum of 4 weeks. A partial response (PR) was defined as a >50% reduction in the product of the perpendicular diameters of the indicator lesions, without the appearance of new lesions. Progressive disease (PD) was defined as an enlargement of >25% in an indicator lesion or the development of new lesions and stable disease (SD) was defined as failure to meet the criteria for response or progression. The adverse events were graded during each treatment cycle using the CTCAE version 4.0. In the event of toxicity, chemotherapy was postponed until the symptoms resolved.

### Survival analysis

The lengths of OS and progression-free survival (PFS) were measured from the initiation of the second-line treatment to death and progression, respectively. The Kaplan-Meier method was used to calculate survival rates.

The primary endpoint of this study was RR and the secondary endpoints were OS, PFS, adverse effects and third-line chemotherapy performance rate. P≤0.05 was considered to indicate a statistically significant difference. A statistical calculation was conducted using the Dr. SPSS II software for Windows.

## Results

### Patient characteristics

The demographic characteristics of the 18 patients enrolled in this study are shown in Table I. The patients were assessed for response and toxicity. The median patient age was 73 years (range, 53–75 years); 16 patients were male (88.9%) and 2 were female (11.1%), with a good overall general condition (Karnofsky performance status: 80–100). The patients had histologically confirmed adenocarcinoma (11 differentiated and 7 undifferentiated).

The first-line treatment was administered in all 18 patients (17 received S-1 and 1 received S-1 plus CDDP), followed in all cases by the second-line treatment, with an average of 3.2 courses (range, 1–7). A third-line treatment was performed in 13 cases (72.2%).

### Efficacy

Assessable lesions were present in all cases. During the second-line treatment, 2 cases of CR, 1 case of PR and 7 cases of SD were identified. The RR was 16.7% and the disease control rate (DCR) was 55.6% (Table II).

### Survival

**Figure 1.**
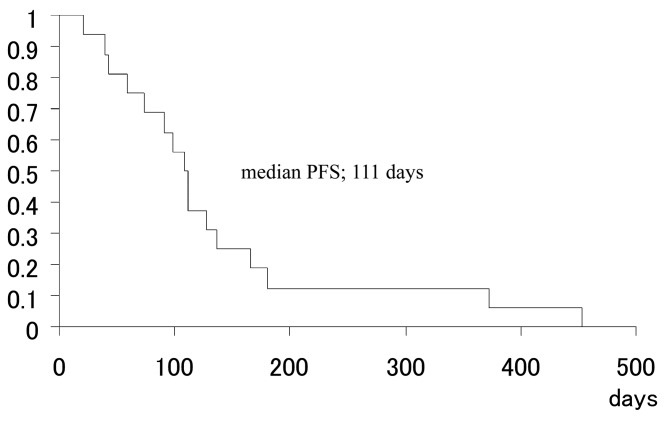
Progression-free survival (PFS) curve (n=18). The median PFS was 111 days (range, 21–749days).

**Figure 2.**
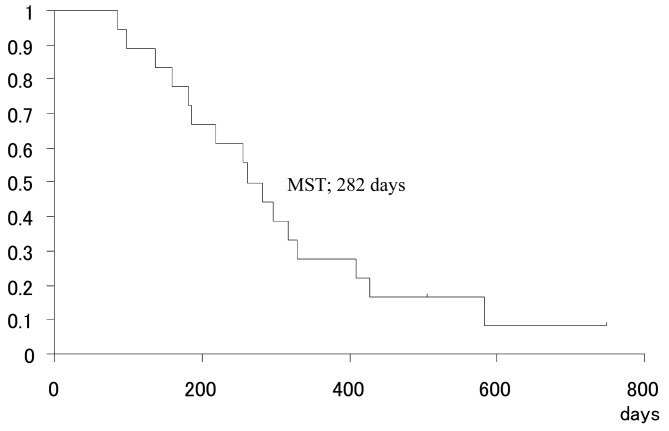
Overall survival curve (n=18). The median survival time (MST) was 282 days.

The mean follow-up time was 271.5 days (range, 85–749 days) and the median PFS was 111 days (range, 21–749 days) (Fig. 1). The median survival time (MST) was 282 days (Fig. 2).

### Toxicity

With regards to hematological toxicity, leukopenia and neutropenia, a side-effect of ≥grade 3 severity, were the most frequently encountered, followed by anemia. Four cases of leukopenia and neutropenia (22.2%) and 2 cases of anemia (11.1%) were identified. With regards to non-hematological toxicity, diarrhea and loss of appetite were the most frequently observed, followed by fatigue. During the second-line treatment, there were 2 cases of diarrhea and loss of appetite (11.1%) and 1 case of fatigue (5.6%) (Table III).

## Discussion

Previous studies have demonstrated a survival benefit for the treatment of advanced gastric cancer in a first-line setting. The SPIRITS trial reported an OS and PFS of 13 and 6 months, respectively, in the S-1 plus CDDP arm in a first-line setting. Subsequently, 74% of the patients who received S-1 plus CDDP and 75% of those who received S-1 alone, were administered second-line chemotherapy (1). The JCOG9912 trial reported an OS and PFS of 11.4 and 4.2 months, respectively, in the S-1 alone arm and 74% of those patients received second-line chemotherapy (2). Furthermore, the FLAGS global trial reported an OS and PFS of 8.6 and 4.8 months, respectively, in the S-1 plus CDDP arm and only 29.6% of those patients received second-line chemotherapy (5). We reported the results from the first-line chemotherapy with S-1 alone, second-line chemotherapy with S-1 plus CDDP and third-line chemo-therapy with weekly paclitaxel. In this therapy, the RRs were not high; however, satisfactory survival rates were observed and the side-effects were minor. We considered this therapy to be effective due to the smooth transition to the subsequent regimen (6). This suggests that, in certain cases, optimal second-line chemotherapy contributed to the favorable OS observed with first-line chemotherapy.

A previous study by Koizumi *et al* (4) reported that biweekly coadministration of 60 mg/m^2^ CPT-11 and 30 mg/m^2^ CDDP is safe and effective for the management of unresectable advanced or recurrent gastric cancer. The overall RR was 32.5% and it was 53.3% in patients who had not received prior chemotherapy (4). We used this CPT-11 and CDDP regimen as a second-line chemotherapy.

Previous studies have reported that CPT-11 exhibited effectiveness as second-line chemotherapy and that the combination of CPT-11 and CDDP at the outpatient setting appeared promising (7–9).

The Osaka Gastrointestinal Cancer Chemotherapy Study Group conducted a phase II study on the biweekly administration of CPT-11 and CDDP to patients with gastric cancer refractory to S-1 (OGSG 0504 study). According to the intention-to-treat analysis, the overall RR was 28.6%, including 4 cases of CR and 6 cases of PR. The DCR was 65.7%. The most common grade 3/4 toxicities were neutropenia (22.4%), anorexia (14.3%), fatigue (8.6%) and diarrhea (2.9%). The median OS was 450 days. The combination treatment of CPT-11 and CDDP was proven to be feasible and effective. Accordingly, this regimen may be considered as one of the standard second-line treatments for gastric cancer (9). Our RR and DCR rates were 16.7 and 55.6%, respectively, and the median OS was 287 days. These results were lower compared to those of the OGSG 0504 trial. However, our grade 3/4 toxicities were comparable. The discrepancies in the results may be attributed to our limited patient sample compared to that of the OGSG 0504 trial.

Furthermore, Oba *et al* (7) reported that CPT-11 mono-therapy (150 mg/m^2^ on days 1 and 15) offered an advantage over the combination therapy with CPT-11 (70 mg/m^2^ on days 1 and 15) plus CDDP (80 mg/m^2^ on day 1) in the second-line setting for the treatment of advanced gastric cancer, following failure of a fluoropyrimidine-based regimen (7). However, this combination therapy was ultimately proven not to be superior to fluorouracil (2).

Our results suggested that CPT-11 and CDDP combination therapy in a second-line setting is an effective regimen in the treatment of advanced gastric cancer. Additional prospective clinical trials may be useful in developing individualized optimal treatments, providing evidence on the efficacy of molecular-targeted agents and the utility of biological markers for the treatment of advanced gastric cancer in a second-line setting.

## Figures and Tables

**Table I.** Characteristics of enrolled patients.

Characteristics	No. of patients	Percentage
Total number of patients	18	100
Age (years)	73	
Median (range)	(53–79)	
Gender		
Male	16	88.9
Female	2	11.1
Karnofsky performance status 80–100	18	100
Histological type (Japanese classification)		
Differentiated	12	66.7
Undifferentiated	6	33.3
Target lesion		
Unresectable	7	38.9
Recurrent	11	61.1
First-line chemotherapy		
S-1	17	94.4
S-1 + CDDP	1	5.6

CDDP, cisplatin.

**Table II.** Treatment efficacy.

No. of patients	CR	PR	SD	PD	RR (%)	DCR (%)
18	2	1	7	8	16.7	55.6

CR, complete response; PR, partial response; SD, stable disease; PD, progressive disease; RR, response rate; DCR, disease control rate.

**Table III.** Occurrence of adverse events.

Regimen	Grade		
	3	4	3 and 4 (%)
CPT-11 + CDDP (n=18)			
Leukopenia	3	1	22.2
Neutropenia	3	1	22.2
Anemia	2	0	11.1
Diarrhea	2	0	11.1
Loss of appetite	2	0	11.1
Fatigue	1	0	5.6

CDDP, cisplatin; CPT-11, irinotecan.

## Abbreviations:

CPT-11: irinotecan;
CDDP: cisplatin;
5-FU: 5-fluorouracil;
PTX: paclitaxel;
DTX: docetaxel;
AST: aspartate transaminase;
ALT: alanine transaminase;
CTAE: Common Terminology Criteria for Adverse Events;
CT: computed tomography;
CBC: complete blood count;
CR: complete response;
PR: partial response;
PD: progressive disease;
SD: stable disease;
OS: overall survival;
PFS: progression-free survival;
RR: response rate;
DCR: disease control rate;
MST: median survival time
